# Finite Element Analysis of Soft-lined Mandibular Complete Denture and its Supporting Structures

**DOI:** 10.5681/joddd.2012.009

**Published:** 2012-06-06

**Authors:** Katayoun Sadr, Jahangir Alipour, Fateme Heidary

**Affiliations:** ^1^Assistant Professor, Department of Prosthodontics, Faculty of Dentistry, Tabriz University of Medical Sciences, Tabriz, Iran; ^2^PhD Student of Agricultural Machinery, Faculty of Agriculture, University of Tabriz, Iran; ^3^Post-graduate Student, Department of Prosthodontics, Faculty of Dentistry, Tabriz University of Medical Sciences, Tabriz, Iran

**Keywords:** Complete denture, soft liner, finite element analysis

## Abstract

**Background and aims:**

There are many edentulous people with severely resorbed residual ridges and non-resilient lin-ing mucosa that are unable to tolerate occlusal forces during functional and parafunctional movements. Lining the tissue surface of dentures with a flexible material can theoretically distribute and absorb forces with cushioning effect. The aim of this study was to evaluate the effect of a soft liner on stress levels in mandibular complete denture and its supporting struc-tures by finite element analysis.

**Materials and methods:**

A simplified 3-dimensional finite element model of relatively resorbed mandible, mucosa, denture and a soft liner was prepared. Then the model, with and without soft liner, underwent normal vertical and lateral occlusal forces. The stresses were analyzed using the ANSYS 12 software.

**Results:**

Using the soft liner increased stress levels up to 18.5% and 30% in the cortical bone and mucosa, respectively, after vertical load was applied in the incisor region. Application of bilateral vertical load on the molar area increased stress in cortical bone u to 44% and in the mucosa up to 29%. Unilateral loading in the canine area increased stress level in the mucosa up to 63.5%. The highest stress was seen at denture base followed by the cortical bone.

**Conclusion:**

Use of soft liners increased stress in denture supporting structures. Higher level of stress concentration was observed primarily in the denture base followed by the cortical bone.

## Introduction

Many edentulous individuals bear resorbed residual ridges with thin and non-resilient lining mucosa unable to tolerate occlusal forces during functional and parafunctional movements, resulting in complaining about pain during mastication. ^1^ The method used as a solution in these cases is utilization of permanent soft liners under hard acrylic denture bases. Soft liners distribute and absorb loads by a cushioning effect and as a result, decrease the amount of forces loaded on denture supporting structures, decrease pain during mastication, and enable the successful use of prosthesis for the patient. ^2 , 3^

Clinical efficacy of soft liners has been reported in numerous studies. Controlled randomized clinical trials indicate that use of silicone soft liners in mandibular dentures improves the mastication efficacy of patients.^4^ Furthermore, utilization of acrylic soft liners under denture bases decreases complications during the first patient visit after delivery session^5^ and the patients’ satisfaction is significantly higher in mandibular dentures with soft liners compared to hard acrylic bases.^6^

Despite the clinical efficacy of soft liners, there is insufficient information on soft liners’ role in load distribution and absorption in denture supporting tissues because of limitations in study methods.^7^Therefore, finite element analysis can be used to evaluate the destination of loads in underlying mucosa and bone. In all the assessments with this method, elastic properties of soft liners under rapid mastication forces have been evaluated. Finite element analysis findings have revealed that stress increases in bone following soft liner use.^8 , 9^Therefore, use of soft liners in patients suffering from bruxism and clenching could not be helpful. On the other hand, stress amount increases in acrylic denture base with soft liner presence.^10^ Finite element analysis has shown^11^ that modulus of elasticity of soft liner should not be lower than that of the lining mucosa. In other words, for maximum cushioning effect, the modulus of elasticity of soft liner should be the same as that of mucosa, which seems logical because the soft liner compensates the lost thickness of lining mucosa. The findings also indicate that the thickness of soft tissue does not affect stress ratio. As a result, excessive thickness of soft liner is unnecessary and just weakens denture base.^11^ It seems the above findings are contrary to positive clinical effects of soft liners. Therefore, this study aimed to evaluate the stress level in underlying tissues of mandibular denture under occlusal forces with and without soft liners by finite element analysis.

## Materials and Methods

**Figure 1:**
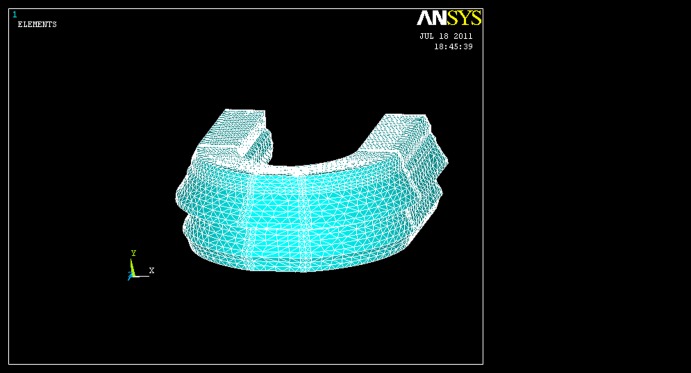
3D and meshed model of mandibular arch with corresponding denture, mucosa and soft liner.

3D simulation of mandibular arch with denture has been performed using an edentulous resorbed mandibular bone and its corresponding denture. In this model, the thicknesses of cortical bone, mucosa, soft liner, and acrylic base were assumed to be 1.5, 1.5, 2, and 3 mm, respectively.^10^ The model was designed by ANSYS software and then 10-point solid92-tetrahedral elements were used for meshing. The prepared model consists of 161707 nods and 113260 elements (Figure 1).

The mechanical properties (elastic modulus and Poisson’s ratio) of different parts of model were taken from literature (Table 1).^7 , 11^Elastic modulus of silicone soft liners stock on the market is between 1 to 20 Mpa. Regarding equality of elastic modulus for soft liner and mucosa, the elastic modulus of 5 Mpa was selected.^7^

**Table 1:** Mechanical properties of materials used in the study

Material	Elastic modulus (MPa)	Poisson’s ratio
Denture base resin	2650	0.30
Soft liner silicone	5	0.49
Mucosa	5	0.49
Cortical bone	13400	0.3
Spongy bone	600	0.3

The model was loaded twice with and without soft liner for simulation of masticatory forces under vertical and lateral movements in the range of normal occlusal forces,^10^consisting of diffused 50-N vertical load on lower incisor region, disseminated 80-N vertical load on mandibular molar region on both sides, and lateral load with 45 degree of angulation and 50 N on mandibular canine region on one side. In all the loading conditions, the model was constrained at the nodes on the interface between the inferior border of mandible and its surrounding mucosa. Then, the prepared model was analyzed with and without the soft liner under three loading conditions by ANSYS software.

## Results

The values of Von Mises stresses for all the conditions are shown in Table 2.

**Table 2:** Von Mises stresses for different layers under various loadings with and without soft liner

Layer	Von Mises stresses (Mpa) with soft liner	Von Mises stresses (Mpa) without soft liner
Vertical load in the incisor region		
Spongy bone	0.081	0.088
Compact bone	2.706	2.281
Mucosa	0.116	0.089
Soft liner	0.062	—
Denture acrylic base	15.432	15.701
Bilateral vertical load in the molar region		
Spongy bone	0.207	0.211
Compact bone	4.052	2.813
Mucosa	0.183	0.142
Soft liner	0.143	—
Denture acrylic base	21.693	21.805
Unilateral oblique load in the canine region		
Spongy bone	0.289	0.290
Compact bone	5.194	5.168
Mucosa	0.175	0.107
Soft liner	0.131	—
Denture acrylic base	37.354	31.374

As described in Table 2, after using elastic soft liner under vertical loading on incisor area, the stress value increased by 18.5% at cortical bone and by 30% at mucosa. In vertical bilateral loading in the molar area, stress increased by 44% and 29% on cortical bone and mucosa, respectively. Unilateral loading with 45-degree angulation on canine area caused 63.5% stress increase on mucosa, respectively. The highest stress was seen at denture base followed by cortical bone.

**Figure 2:**
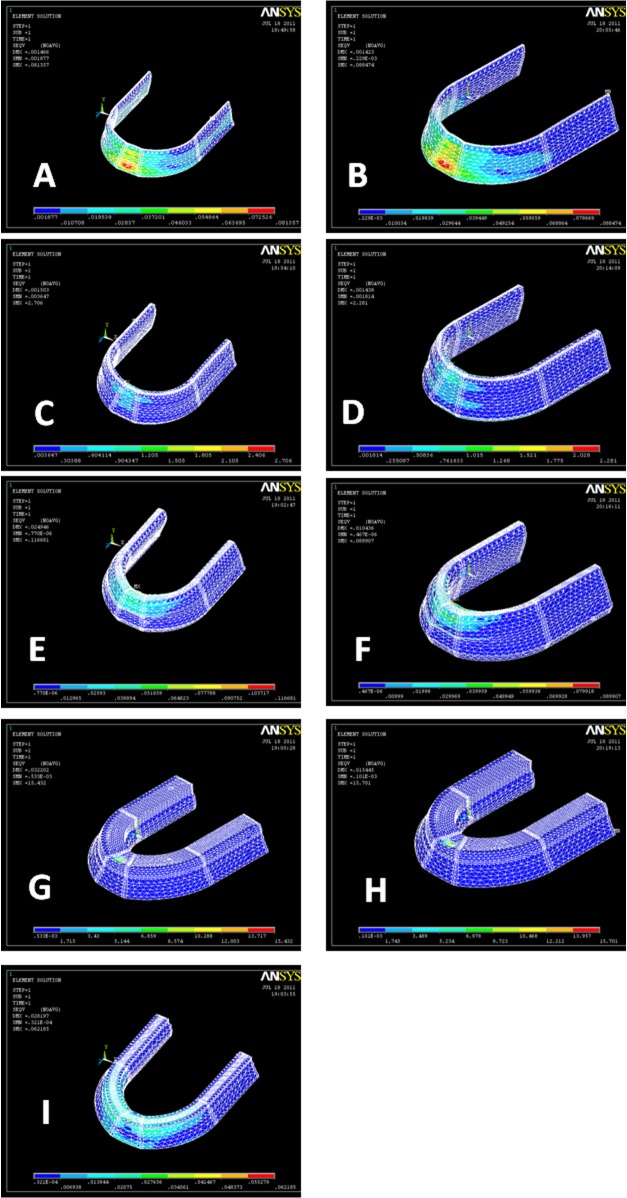
Stress distribution under vertical load in the incisor region within, A: Spongy bone with soft liner; B: Spongy bone without soft liner; C: Compact bone with soft liner; D:Compact bone without soft liner; E: Mucosa with soft liner; F: Mucosa without soft liner; G: Denture acrylic base with soft liner; H: Denture acrylic base without soft liner; I: Soft liner.

**Figure 3:**
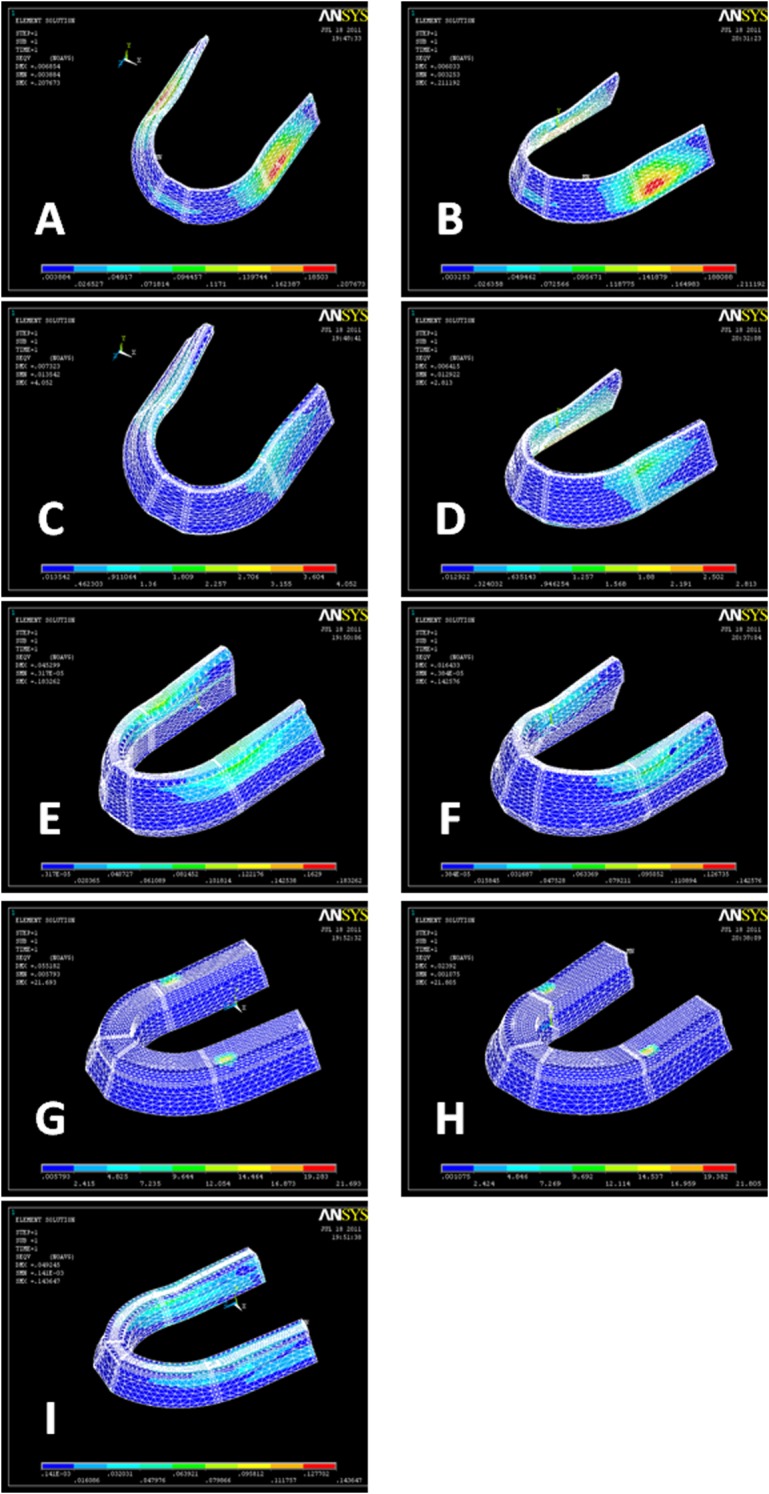
Stress distribution under bilateral vertical load in the molar region within, A: Spongy bone with soft liner; B: Spongy bone without soft liner; C: Compact bone with soft liner; D: Compact bone without soft liner; E: Mucosa with soft liner; F: Mucosa without soft liner; G: Denture acrylic base with soft liner; H: Denture acrylic base without soft liner; I: Soft liner.

**Figure 4:**
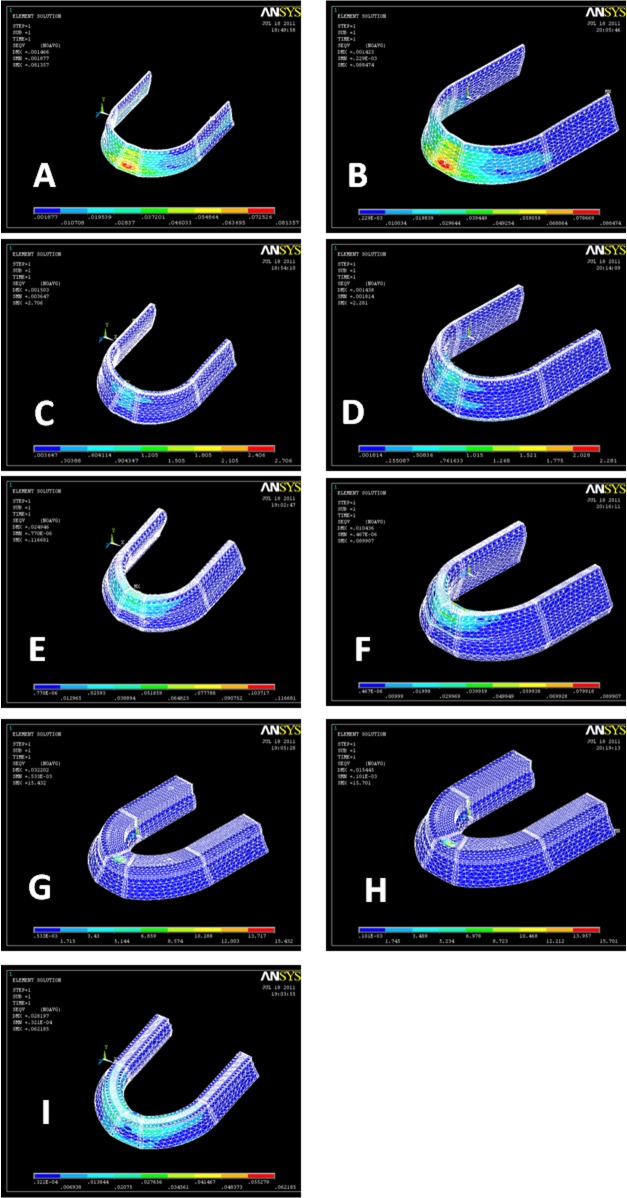
Stress distribution under unilateral oblique load in the molar region within, A: Spongy bone with soft liner; B: Spongy bone without soft liner; C: Compact bone with soft liner; D: Compact bone without soft liner; E: Mucosa with soft liner; F: Mucosa without soft liner; G: Denture acrylic base with soft liner; H: Denture acrylic base without soft liner; I: Soft liner.

Stress distribution within different layers of denture and supporting structures in three different load cases are shown in Figures 2-4.

## Discussion

Although soft liner materials are far from ideal,^10^ clinical evaluations have shown that they improve patients’ masticatory efficacy and satisfaction level and decrease pain during mastication. ^2- 6^ In this study, Von Mises values in soft-lined mandibular complete denture were compared with those of conventional mandibular denture. Research hasshownthat at different loading conditions, the highest amount of stress is seen at acrylic base and cortical bone and when elastic soft liner is used the stress is higher than when hard acrylic base is used. In vertical bilateral loading on the molar region, Von Mises stress on cortical bone increased by 44% when using soft liner, consistent with the results of a study by Balatlioglu et al.^9^Therefore, it was suggested that soft liners should not be utilized in patients with bruxism.^8 , 9^ Increased Von Mises stress in an acrylic base covered with soft liner has also been reported in other studies. Therefore, the fracture risk of denture base increases with the use of soft liners.^9- 11^

Furthermore, the results showed that the Von Mises stress increased on mucosa with the use of a soft liner, contrary to the results of other studies.^9^ In an in vitro study, Taguchi et al^12^ showed that as the thickness of silicone soft liner increases, minor changes in strain would occur compared to acrylic soft liner. As a result, silicone soft liner must transfers more stress to underlying tissues. On the other hand, elastic properties of soft liner should be similar to mucosa to obtain optimum cushioning effect.^1 , 6 , 11^Therefore, in the this study, soft liner with a thickness of 2mm and mucosa with a thickness of 1.5 mm had the same mechanical properties. The presence of 3.5-mm-thick elastic layer, that could transmit the major part of occlusal forces, can be attributed to stress increase in underlying mucosa and bone.

Although based on the results of this study the stress increased on cortical bone and mucosa using silicone soft liners, why do patients using silicone soft liners report more satisfaction compared to those using acrylic base?^1^ In response, we can propose a hypothesis regarding the fact that use of soft liner prevents the friction between the base and the mucosa and pain by increasing denture base retention and stability and decreasing its movements.^3 , 13^ Furthermore, increased denture retention and stability provides the successful use of denture during function and increases its masticatory efficacy for the patient. All the above-mentioned factors could increase patient satisfaction without having any effect on stress distribution.

This study also showed that the oblique load can create maximum Von Mises stress value under all loading conditions. This finding indicated the importance of minimizing lateral forces and elimination of premature occlusal contacts by proper selection of occlusal scheme in complete dentures.

In order to confirm the above-mentioned hypothesis, comparison of stresses developed in denture supporting tissues following the use of acrylic and silicon soft liners should be carried out and also clinical effects of using elastic soft liners in denture supporting tissues should be evaluated.
